# Trained innate immunity, epigenetics, and food allergy

**DOI:** 10.3389/falgy.2023.1105588

**Published:** 2023-05-26

**Authors:** Llilian Arzola-Martínez, Catherine Ptaschinski, Nicholas W. Lukacs

**Affiliations:** ^1^ Department of Pathology, University of Michigan, Ann Arbor, MI, United States; ^2^ Mary H. Weiser Food Allergy Center (MHWFAC), University of Michigan, Ann Arbor, MI, United States

**Keywords:** food allergy, trained immunity, microbiota, epigenetic, metabolic reprogramming

## Abstract

In recent years the increased incidence of food allergy in Western culture has been associated with environmental factors and an inappropriate immune phenotype. While the adaptive immune changes in food allergy development and progression have been well-characterized, an increase in innate cell frequency and activation status has also recently received greater attention. Early in prenatal and neonatal development of human immunity there is a reliance on epigenetic and metabolic changes that stem from environmental factors, which are critical in training the immune outcomes. In the present review, we discuss how trained immunity is regulated by epigenetic, microbial and metabolic factors, and how these factors and their impact on innate immunity have been linked to the development of food allergy. We further summarize current efforts to use probiotics as a potential therapeutic approach to reverse the epigenetic and metabolic signatures and prevent the development of severe anaphylactic food allergy, as well as the potential use of trained immunity as a diagnostic and management strategy. Finally, trained immunity is presented as one of the mechanisms of action of allergen-specific immunotherapy to promote tolerogenic responses in allergic individuals.

## 1. Introduction

Food allergy prevalence in western society has increased in the last several decades and is considered part of the “second wave” of the allergic epidemic (1). The increment in the number of worldwide cases have been associated with changes in lifestyle and modernization (cesarean delivery and western diet), access to antibiotics, urbanization and industrialization (2). Different hypotheses posit that these socioeconomics changes directly affect immune development through pathogen exposure (hygiene hypothesis) (3, 4) or that they are indirectly impacting immune development through the development of leaky epithelial barrier in the skin, airways, and gut mucosa (barrier hypothesis) (5, 6) resulting in microbiome dysbiosis, inappropriate immune responses and allergic diseases development.

**Figure 1:**
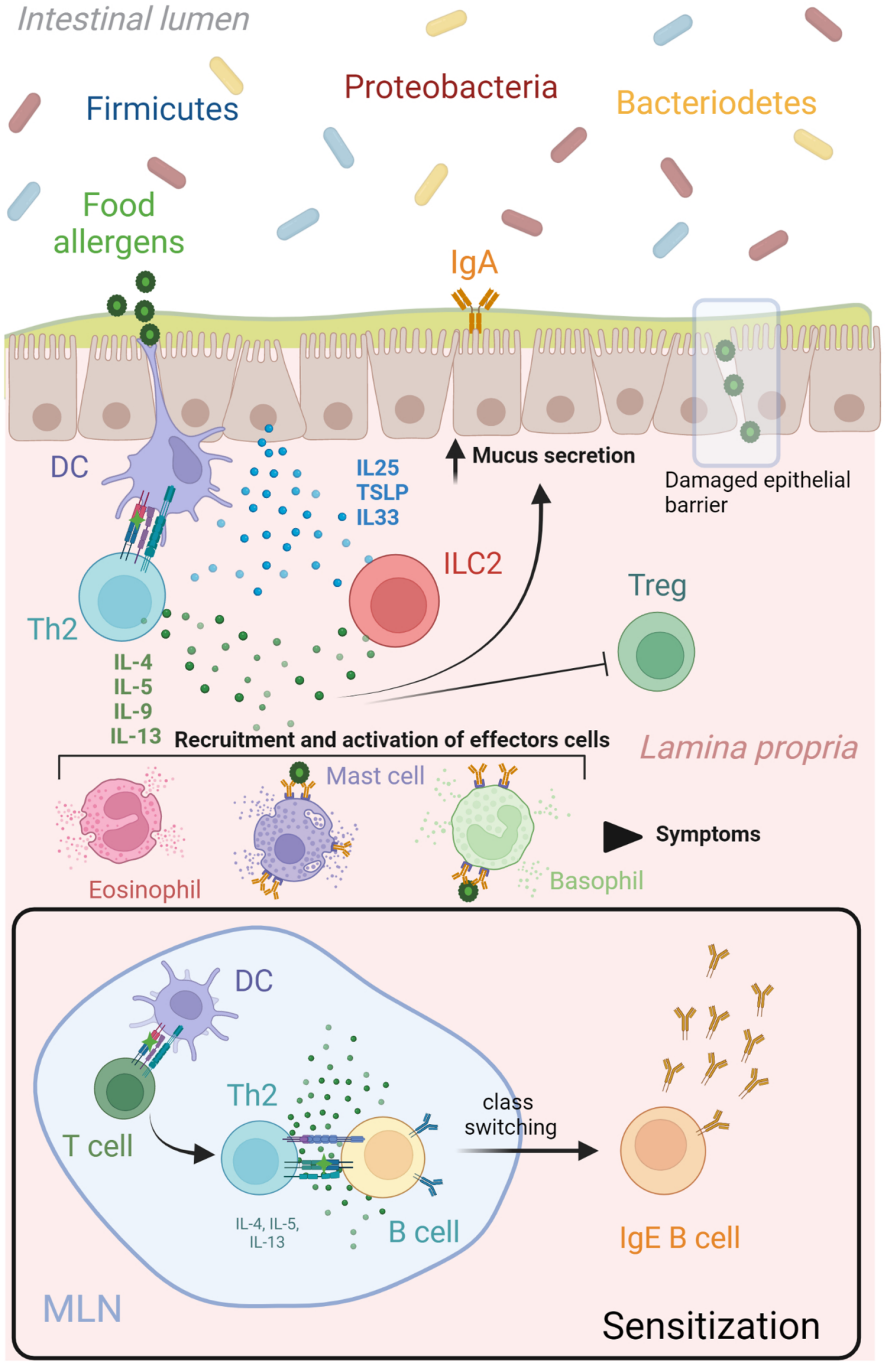
Th2 network in food allergy. During the sensitization phase, mature dendritic cells migrate to lymphoid organs to present the uptake food allergen to naïve T cells. A clonal expansion process is triggered resulting in the differentiation of allergen-specific Th2 cells that produce cytokines (IL4, IL5, IL9, IL13) and lead many aspects of allergic inflammatory responses. These Th2 derived cytokines (IL4 and IL13) initiated an IgE class switch recombination in naïve B cells, differentiation of plasma cells and allergen-specific IgE production. A subsequent encounter of dendritic cells with the same food allergen at the epithelial barrier will drive a type 2 immune response characterize by increased mucus and Th2 cytokines production, as well as recruitment and activation of effector cells (mast cells, basophils, eosinophils). This Th2 immune cascade is potentiated by the release of alarmins (IL25, IL33 and TSLP) from epithelial cells after damaged or been stressed by allergens, which upregulate stimulatory signals for T cell activation in dendritic cells and activate production of Th2 cytokines from ILCs. Food antigens that pass the epithelial barrier can bind to crosslinked allergen-specific IgE-FcεRI molecules on mast cells and basophils, leading to release of inflammatory mediators that produce the symptoms associated to a food allergic response. DC, dendritic cell; IL interleukin; ILC, innate lymphoid cell; MLN, mesenteric lymph node; TSLP, thymic stromal lymphopoietin. Created with Biorender.

The pathophysiology of food allergy is characterized by a poorly controlled/amplified Th2 inflammatory immune response to otherwise innocuous antigens (7). This type 2 immune response can provide protection from helminths, but is harmful in maintaining gut homeostasis in the context of food allergens (8). The mechanisms of allergic sensitization and response have been comprehensively reviewed elsewhere (7, 8) and involves an intricate crosstalk between structural and immune cells from both adaptative and innate arms of the immune system (Figure 1) (1, 8). The classical epitope-specific adaptive immunological memory has been widely explored in food allergy (9). In addition, an altered innate immune signature characterized by increased number and function of innate immune cells is present in food allergic patients early in life and can persist into adulthood (10, 11). However, the application of the new concept of trained innate immune responses in food allergy is just beginning to be investigated (12). Here we explore the studies done on the adaptability of the innate immune system in food allergy. While “food allergy” as an umbrella term encompasses many disease types, this review will focus on IgE-mediated, mast cell-driven anaphylactic allergic responses.

## 2. Trained immunity

The innate immune system has long been defined as non-specific and lacking recall response, whereas the adaptive immune system forms memory responses against specific antigens. However, recent evidence has found that the innate immune system can indeed have “memory-like” features that program innate immune cells to respond based upon transcriptional activation. This phenomenon, termed “trained immunity,” generates an “educated” response to a subsequent encounter with a similar/distinct stimulus that activates common pathways. Epigenetic modifications and metabolic changes are two critical underlying mechanisms of long term trained innate immunity (13). The priming of the innate cell may result in a faster and stronger immune response, or conversely may lead to an attenuated, tolerogenic response (14, 15). The balance of these two contrasting program effects allows the innate immune system to respond appropriately in health and disease (16, 17). Understanding how to drive these different innate immune programs could be key to maintaining homeostasis and avoiding disease.

Recent studies have shown that protective innate immune responses can be stimulated by several well-known agents, including *Bacillus* Calmette-Guérin (BCG) vaccination (18), β-glucan (19, 20) and *Candida albicans* (14). Others exposures such as LPS (21), peptidoglycan (22), herpes virus (23), parasitic helminth (24, 25), and metabolic stimuli (26, 27) can also influence the development of trained immunity-like responses. The immunological phenotype associated with this programming can last for several months to years in individuals (28–30), often reinforced by continued or similar environmental stimuli. A variety of innate immune cells from the myeloid (monocytes, macrophages, dendritic cells, neutrophils and eosinophils) or lymphoid (natural killers [NK] cells, Innate lymphoid cells [ILC] 1 and 2) lineage go through epigenetic and metabolic reprogramming after bacterial, helminth and fungal infections, allergen stimulation, or vaccination (17, 19, 31, 32). Intriguingly, the life span of innate immune cells is relatively short (33), but recent studies have demonstrated that myeloid hematopoietic stem cells in the bone marrow (20, 34) and epithelial stem cells at the respiratory mucosal barrier (35) similarly develop a trained phenotype and stand ready to replenish immune and non-immune cells populations. Non-immune cells with a longer life span such as intestinal stromal cells, mesenchymal stromal cells, microglia, and fibroblast also exhibit epigenetic reprogramming that can contribute to the development of immune responses through production of mediators that affect local environments (36). In addition, the innate immune programing may be passed on *in utero* (37). Evidence suggests that prenatal maternal pathogen infections can shape the ultimate immune landscape in offspring, by changing T cell populations and altering the cytokine profile (38–40). Notably, BCG maternal vaccination status has a positive effect on child survival associated to the beneficial nonspecific effects of this live vaccine (41). In addition, maternal microbiome has arisen as one of the environmental factors that influences the vertical transmission of allergen-induced trained immunity (42). Overall, numerous studies provide evidence that maternal trained immunity could determine long-term immunological responses by modifying cellular phenotypes in offspring generations.

### 2.1. Trained immunity in food allergy

In the last several years, environmental and lifestyle factors have highly impacted the etiology of food allergy (7). Although trained immunity can result in an effective immune defense against infections, it can also trigger a hyper-inflammatory response that worsens an established inflammatory condition (16, 17) or contributes to the onset of a new pathological state (43). Some authors have associated food allergy with excessive trained immunity (43), and the loss of immune tolerance to food allergens early in life (44) or later in adulthood (45). In addition, food-allergen exposed allergic individuals often have a hyper-inflammatory innate immune response (12). Therefore, these associations suggest that trained immunity mechanisms are implicated in the development and persistence of food allergy. The role of trained immunity in the establishment of an allergic (Th2) vs. a tolerizing phenotype is not fully understood but it has been reported that changes in the abundance or functional status of crucial cells can influence local and systemic immune responses in food-sensitized individuals. The development of food allergy later in life was linked with an exaggerated inflammatory cytokine response after toll like receptor (TLR) stimulation in cord blood derived-mononuclear cells from allergic children compared to non-allergic controls in two birth cohorts (10, 46). The follow-up of one cohort showed that TLR responses were attenuated by the age of 5 years in the allergic subjects, which points to an abnormal early-stage maturation of the innate immune system in these children (10). The incubation of naive CD4+ T cells from cord blood coming from allergic donors with IL-1β+TGF-β and TNF-α+TGF-β cytokines resulted in an amplified type 2 immune responses (46). This experiment confirmed the association between early life innate immune hyperresponsiveness and predisposition to develop type 2 immune responses with ageing. An increase of the monocyte population in the cord blood was also associated with predisposition to develop allergen-specific type 2 responses (46). Another study found that monocytes from egg-allergic infants had an enhanced innate immune response to TLR stimulation and lower number of CD4+ regulatory T cells (47). In addition, a different cohort of challenge-confirmed egg-allergic 1-year-old infants showed increased numbers of circulating monocytes and dendritic cells compared with nonallergic controls. Also, cytokine production from sorted CD3-depleted cells (non-T cell fraction) was characterized by a high baseline concentration of the inflammatory cytokine IL-8, and lower production of Th1-associated (IL-12p70) and regulatory (IL-10) cytokines. These cytokines were further increased when cell from allergic patients were stimulated with LPS, suggesting the presence of a trained innate inflammatory response. The follow-up (at 2–4 years old) showed that infants who naturally develop clinical tolerance have a significant increase in the number of circulating myeloid DCs from baseline, while individuals with persistent egg allergy had an increased presence of monocytes (11). Combined, these studies suggest that innate immune cells population from food allergic individuals exhibit trained immunity features such as an increased myeloid circulatory population and hyperinflammatory phenotype (17), which may contribute to the DCs:T cell interaction and skew the balance in favor to allergic type 2 immune responses (48).

Future studies in food allergy need to be done describing other important cellular contributors to the allergic type 2 immune response. For example, ILC2s are a major source of IL-4, IL-5 and IL-13 cytokines that drive type 2 allergic inflammation by regulating (i) IgE production by B cells, (ii) mediating eosinophil differentiation, recruitment, and activation, and (iii) promoting mucus production and tissue remodeling in the epithelial cell layer, respectively (49). The activation of ILC2s is indirectly mediated by allergens and regulated by epithelial cell-derived cytokines including IL-25, thymic stromal lymphopoietin (TSLP), and IL-33, as well as lipids and neural signals (50). In a recent study using a peanut sensitized mouse model, it was shown that IgE-activated mast cells drive intestinal ILC2s expansion and IL-13 production in an IL-4Rα-dependent manner. In turn, ILC2 activation increased sensitivity to mast cells mediators leading to an amplified systemic anaphylaxis (51). Additionally, the tuft cell–ILC2 immune signaling loop in the small intestine has been proposed to amplified food allergy related anaphylactic symptoms (52). This circuit is initiated by the production of IL-25 by epithelial tuft cells (also called brush cells) which induce IL-13 production by ILC2s located in the lamina propria (53, 54). IL-13 production leads to epithelial remodeling marked by goblet and tuft cell hyperplasia, that feed-forward to ILC2s activation (53, 54). Other Th2 cytokines produced by ILC2s, such as IL-4 stops the production of regulatory T cells (55) and promotes intestinal mast cell accumulation resulting in IgE-mast cells activation and increased gut permeability worsening allergic responses (56). The accumulation of secretory cells such as tuft cells also contribute to food induced anaphylactic response by promoting secretory-antigen passages in the small intestine, facilitating food allergens to cross the epithelial barrier and contact IgE inducing mast cell degranulation (57). In addition, tuft cells are considered epithelial chemosensory cells specialized in monitor microbial metabolites, such as succinate. Microbiome modification after deleterious environmental exposures have been associated with elevated levels of succinate in the lumen, which boosts type 2 immunity mediated by tuft cells, predisposing the host and increasing the reaction severity to food allergens (52).

While less is known about the innate training of cells such as ILC2s and epithelial cells in the intestine, more work has been done to understand these cells and their role in the development of Th2 responses in the airways. In the lung, ILC2s are trained by exposure to allergens and IL-33. These allergen-primed ILC2s acquired non-specific memory-like type 2 immune responses, that could explain the response to multiple allergens in asthmatic individuals (49, 50). On the other hand, the presence of retinoic acid and IL-33 direct the generation of immunosuppressive trained ILC2s that produce IL-10 cytokine, showing the importance of the local cytokine environment to regulate immune responses in these cells (50). In the same way, it was shown that *ex vivo* cultures of basal progenitors of airway epithelial cells conserve intrinsic memory of IL-4/IL-13 exposure, contributing to the disruption of the epithelial barrier and persistence of allergen-driven type 2 inflammation in the airway (35). Other cellular players in type 2 inflammatory responses, such as macrophages (58) and eosinophils (59, 60) have been reported to also undergo innate training after challenge with allergen and parasite infection. Furthermore, macrophages experienced metabolic and epigenetic reprograming that contributes to the chronic type 2 airway inflammatory landscape in nonsteroidal anti-inflammatory drug-exacerbated respiratory disease (N-ERD) (61). Determining whether similar trained innate immune responses occur in the gut will be critical to understanding the potential to develop therapeutic targets and preventative approaches to food allergy.

## 3. Epigenetic and metabolic reprograming in food allergy

The potent transcriptional response to allergens that characterize innate trained immunity (15) is accompanied by epigenetic regulation of genes related to immune function, chromatin remodeling and alteration of overall metabolic activity (13, 62). These two mechanisms (epigenetic and metabolic) work together to fine-tune the appropriated innate immune response.

### 3.1. Epigenetics

While genetic variants associated with food allergy still need to be carefully studied, it is clear that genetic influence in food allergy has a dominant role. Candidate-gene and genome-wide association studies (GWAS) have revealed new and old correlations and a diversity in the loci variants (polygenetic) associated with food allergy (63–65). However, the variety of food allergy phenotypes, clinical manifestations and responses cannot be only attributed to genetic predisposition. This immune disorder is a result of a complex interplay between the gene pool and exposure to different environmental factors (66–68). Epigenetic mechanisms can mediate the bidirectional communication between genes and environment and therefore contribute to the disease development and course (66). Preliminary evidence suggested that the exaggerated Th2 immune response, commonly share by allergic diseases, is tightly regulated by epigenetic mechanisms that promotes the immediate release of Th2 cytokines in response to allergens, and the heritability of the Th2 phenotype (69).

The epigenetic modification of the chromatin reversibly regulates gene transcription without altering the nucleotide sequence in the genome (66). In general, the control of transcriptional programs at the epigenetic level can be exerted by three different mechanisms. Two of them involve the addition or removal of biochemical groups by enzymes, directly modifying the DNA (methylation) or the tail of core histones (acetylation, methylation, phosphorylation, biotinylating, etc.) (70). DNA methylation involves the addition of methyl groups to cysteine in CG rich regions, which is instructive for transcriptional silencing (71). Lastly, small, non-coding RNA molecules known as microRNAs (miRNA) are another mechanism of epigenetic modification and does not involve enzymatic regulation. Instead, miRNA molecules are involved in the post-transcriptional regulation of gene expression during allergic immune responses in epithelial and immune cells and act by binding to the 3′ untranslated region of an mRNA molecule, thereby destabilizing the mRNA and leading to its degradation (72). Together, the epigenetic machinery is used to stabilize cellular “memory” and influence in the cellular fate and identity (73). Given the relatively sudden rise in allergy in general and food allergy specifically, the changes in environmental epigenetic modification of immune gene transcription is a logical mechanism leading to enhanced disease incidence. For example, early-life differential DNA methylation patterns in allergic individuals is currently being investigated as a potential childhood biomarker (74).

Research on the epigenetics of food allergy is very limited. Only a few studies have shown that the immune response in food allergy is under epigenetic regulation. DNA methylation patterns in childhood food allergy is by far the most studied epigenetic mark. Previously, changes in DNA methylation were connected to aberrant gene transcription and enhanced risk of developing allergic airway disease (75). Recent studies have suggested DNA methylation as a potential epigenetic mechanism that may be diagnostic and could direct therapeutic intervention in food allergy (76, 77). To date, numerous studies have looked at the role of DNA methylation in allergic and tolerogenic responses in humans and in mouse models. These studies have focused on differential DNA methylation patterns in the promoter regions of genes associated with Th1 and Th2 cells, as well as on regulatory T cells. For example, Canani et al. (78) explored the link between DNA methylation and gene expression in peripheral blood mononuclear cells of children with active IgE-mediated cow's milk allergy, tolerant children, and healthy controls. They found opposing methylation patterns in the promoter regions of Th1 and Th2 cytokines genes in children with active IgE-mediated cow's milk allergy compared to tolerant children and healthy controls. In allergic subjects they found a correlation between the promoter methylation pattern and serum cytokine expression. In addition, Petrus et al. studied a cohort of Dutch children and found that the hypermethylation in the cow's milk allergic group was reestablished to control levels in allergic children who develop tolerance, indicating a potentially transient/reversible nature of epigenetic modification (79). The *FoxP3* gene, that codifies a member of the forkhead transcription factor family, is epigenetically regulated in Treg cells (Treg) during food allergy (46, 80, 81), being less transcriptionally active in allergic children (80, 82). Thus, specific regulation of gene expression by DNA methylation appears to correlate to food allergic disease development. These studies and others involving DNA methylation of T cell responses in food allergy are summarized in Table 1. Although most of the studies explore DNA methylation profile as a promising biomarker, other mechanisms of epigenetic regulation are also implicated in food allergy. In one study, the acquired tolerance associated with raw milk consumption in a murine model of OVA-induced food allergy was linked to a lower histone acetylation of Th2 genes in splenocyte-derived CD4^+^ T cells (83). Furthermore, the protective effect of early life consumption of raw cow's milk and breastfeeding on the development of allergic disease have been associated with other epigenetic mechanisms, such as miRNA, contained in extracellular vesicles or exosomes from human and cow's milk (84). miRNA have been implicated in the allergic and anti-helminthic immune gene regulation in Th2 cells (85) and the induction of Treg function (86, 87) during the preventive response to allergy. However, miRNA gene regulation is still an epigenetic mechanism largely unexplored in food allergy. So far, a total of 16 miRNA have been found to be differentially regulated in peripheral blood mononuclear cells obtained from cow milk allergic children compared to healthy controls. Specifically, miR-1931-5p was the most downregulated miRNA in allergic children and was found to be a post-transcriptional regulator of *IL4* (88). Thus, miR-1931-5p was proposed to be a novel diagnostic and therapeutic target for cow milk allergy in infants.

**Table 1:** DNA methylation of T cells in allergic diseases.

Hypermethylation of promoter regions of Th2-associated genes in allergic mice, and hypomethylation in animal model of immunotherapy	Song et al. 2014, Mondoulet et al. 2015, Mondoulet et al. 2019
Signature methylation patterns early in T cell differentiation in pediatric food allergic patients indicating a Th2 bias	Martino et al. 2014; Martino et al. 2018, van Panhuys, Le Gros, and McConnell 2008
Opposing methylation patterns of promoter regions in children with IgE-mediated cow's milk allergy, with decreased methylation of *IL4* and *IL5* and increased methylation of *IFNG* and *IL10*.	Berni Canani et al. 2015, Hong et al. 2016, Acevedo et al. 2021
Hypermethylation of *FOXP3* in allergy, leading to increased allergic responses	Bacchetta, Gambineri, and Roncarolo 2007, Petrus et al. 2016
Hypomethylation of *FOXP3* in tolerogenic responses in peanut OIT	Syed et al. 2014, Petrus et al. 2016, Wang et al. 2018

While the focus of most studies has been on the T cell responses, there are several other targets that are likely very relevant. B cells are another component of the acquired immunity that can be epigenetically modified in food allergy. These cells produce allergen-specific immunoglobulins such as IgE that mediates hypersensitivity reactions, having an important role in the allergic responses (89). The epigenomic and transcriptomic profiles of B cells purified from adolescent patients with peanut allergy, multi-food allergy, and non-allergic controls showed differences in the methylation pattern and gene expression that differentiates a food allergic individual from healthy controls. In addition, peanut allergy and multi-food allergy patients had group-specific epigenetic modifications in promoter regions recognized by transcription factors that regulated B- and T-cell development, B-cell lineage determination, and TGFβ signaling (90). This study highlights epigenetic modifications as a promising technique to distinguish between individuals with single-food allergy and multi-food allergy. Further epigenetic studies in food allergy-related innate immune cells are scarce, although it has been found that mast cell activation during the effector phase of a food allergy immune response can be regulated through altered histone acetylation (91). The pretreatment of bone marrow derived mast cells with the histone deacetylase inhibitor trichostatin A (TSA) *in vitro* decreased transcriptional activity of key genes involved in the degranulation and inflammation process including *FcεRI*, *Il4*, *Il6*, *Il13* and *Tnfα*. The treatment of OVA-induced food allergic mice with TSA attenuated the development of food allergy symptoms and mast cell activation (91). In a recent study that examined epigenetic regulation, authors observed significant decreases in DNA methylation levels for the genes *IL1β* and *IL6* in peripheral blood mononuclear cells of peanut allergic patients compared with non-allergic individuals (92). They also found increased secretion of proinflammatory cytokines in food-allergic compared with non-allergic participants after stimulation.

Epigenetics in food allergy is still a field in the early stages of development. Additional descriptive and functional studies may help to improve our understanding of gene expression regulation by epigenetic mechanisms and how this expression patterns may correlate with clinical outcomes in the context of food allergy. Modification of the epigenetic landscape is a promising novel strategy for prediction, prevention, and treatment of this immune disease.

### 3.2. Importance of microbiota and probiotics to shape the epigenome in food allergy

While genetics can predispose individuals, environmental factors appear to participate in the development and/or maintenance of allergic disorders (67, 68). The diversity of microorganisms colonizing the surface and cavities of the host body is a major environmental factor that can shape the epigenome (93). These commensal bacteria use bioactive molecules produced by microbial metabolism (short chain fatty acid, vitamins, polyphenols, polyamines) and structural components (LPS, peptidoglycan, flagellin) as messengers to interact with the host local (monocytes/macrophages and NK cells) or systemic (hematopoietic progenitors in bone marrow) immune cells (94). It has been proposed that this direct/indirect communication could change the immune cells' epigenetic and metabolic programs and enhance their response to subsequent antigen stimulation (94). An imbalance in the composition and/or function of the gut microbiota, called dysbiosis, has been found to be detrimental for the commensal interaction between host immune system and microbes (95), and led to inflammatory disorders such as food allergy (96). Early life probiotic supplementation has emerged as a useful strategy to shape the gut microbiota and to prevent the development of allergic diseases (95). In a mouse model, prenatal and postnatal consumption of probiotic fermented milk containing the probiotic bacteria strain *Lactobacillus casei* DN-114001 positively change the bacterial colonization pattern in the newborn and stimulated their mucosal immunity (97). Cortes-Perez N. et al. investigated the ability of probiotics to induce trained immunity. They associated the systemic protective effects related to probiotic oral consumption with trained innate immune cells reaching other sites of the common mucosal system, as well as translation of microbial components to bone marrow where they can stimulate myelopoiesis and long-term immune effects (98). However, the desired immunological response depended on the selection and combination of probiotic strains, as different strains can drive either proinflammatory or anti-inflammatory responses (99). Mainly lactobacilli and bifidobacterial lactic acid bacteria have been tested to determine whether probiotics can be used in the prevention of allergies (95). However, conflicting results have prevented them from being systematically used for the prevention of food allergy (95).

A few studies have explored the impact of probiotics on epigenetics and host immunity in the context of food allergy. Differential epigenetic modulation was exerted by a variety of dietary interventions in IgE-mediated cow's milk allergy in children. The patients receiving a hydrolyzed casein formula with the probiotic *Lactobacillus rhamnosus* GG showed a differential DNA methylation rate in *IL4*, *IL5*, *IL10*, and *IFNγ* compared with children that received soy formula (100), reverting to the methylation profile observed by Canani et al. in cow's milk allergic children (78). Also, the probiotic administration differentially modulated microRNA expression, including miR-155, -146a, -128 and -193a (100), all of which can have immune altering effects. Another study, in which children with cow's milk allergy were also supplemented with the hydrolyzed casein formula containing the probiotic *Lactobacillus rhamnosus* GG, an upregulation of the demethylation rate on *FoxP3* gene was found, indicating that this probiotic was able to enhance tolerogenic Treg function (80). Peripheral mononuclear blood cells incubated *in vitro* with the protein fraction of the extensively hydrolyzed casein formula also increased the DNA demethylation rate of the *FoxP3* gene, as well as IL10 and IFNγ production and increased CD4+ FOXP3+ Treg number (101). Both investigations associated the development of cow's milk tolerance, with changes in the methylation epigenetic profile in the *FoxP3* gene (80, 101). In addition, the *Lactobacillus rhamnosus* GG supplemented formula increased butyrate-producing bacterial strains in the gut of food allergic infants (102). The metabolite butyrate can inhibit histone deacetylase activity (103), which stimulates the production and function of regulatory T cells (104). Together, these studies suggest the ability of probiotics to promote a tolerogenic environment by modifying cell immunological program and responses.

### 3.3. Metabolism

The epigenetic and transcriptional reprogramming inherent to trained innate immunity is also accompanied by metabolic alterations (105). During trained immunity the interaction of microbial ligands to specific receptors can result in the differential activation of cellular metabolic pathways (43). The innate cells' energetic metabolic reprograming includes glucose metabolism (glycolysis, oxidative phosphorylation, TCA cycle and pentose phosphate), cholesterol (mevalonate), fatty acid (synthesis and β-oxidation) and glutaminolysis pathways (13, 43). A cellular-specific energetic metabolic shift between oxidative phosphorylation (OXPHOS) and glycolysis facilitates the activation of innate immune cells and their ability to function as trained cells (13, 16). For trainable cells such as macrophages, retaining metabolic diversity is crucial to effectively respond to secondary challenges and depends on the stimulus used to activate/polarize these cells. An elegant study from Lundahl et al. demonstrated that acute alternative activation with Th2 cytokines (IL-4 and IL-13) programs bone marrow-derived macrophages to enhance pro-inflammatory cytokine secretion and killing capacity in response to a secondary mycobacterial infection. These trained macrophages shifted towards a pro-inflammatory cytokine profile previously associated with classically activated macrophages but retain OXPHOS metabolism characteristic of alternative activation, suggesting that OXPHOS can be important for pro-inflammatory responses (106). Other authors pointed out that alternative activated macrophages maintain their metabolic versatility (107), crucial for skewing towards an anti-mycobacterium response. In addition, proinflammatory cytokine secretion by trained macrophages was abrogated by inhibition of DNA methylation, although epigenetics changes were not fully addressed by the authors in this investigation (106).

Nevertheless, little is known about the metabolic reprogramming of innate immune cells in a Th2 driven environment such as food allergy. A study that investigated naïve CD4+ T cell activation in a pediatric cohort of egg allergy subjects compared to non-atopic controls found that pathways related to fatty acid metabolism (cholesterol homeostasis) were significantly upregulated in allergic individuals (108). These authors also detected changes in DNA methylation in genes involved in the metabolic regulation of oxidative phosphorylation and glycolysis (*RPTOR, PIK3D, MAPK1, FOXO1*) which were upregulated in the allergic children (108). Hong et al. used KEGG database to analyze more than 300 genes that had at least one differentially methylated position associated with milk protein allergy in whole blood samples. They identified three enriched metabolic pathways related with carbohydrate metabolism (starch & sucrose metabolism, fructose and mannose metabolism) and antibiotic biosynthesis (butirosin and neomycin biosynthesis), that shares hexokinase genes important for the glucose metabolism (109). Finally, Khader et al. showed that accumulation of substrates of the tricarboxylic acid cycle (succinate and fumarate) can indirectly impact the histone methylation through the inhibition of demethylase activity (110). These results support previous suggestions that epigenetic changes and metabolism are reciprocally regulated (111) and crucial for the development of a trained phenotype.

## 4. Trained immunity as a therapeutic strategy in food allergy

There is contrasting evidence that trained immunity mediates a deleterious effect and atypical innate immune responses after exposure to stimuli in food allergy (47), and it is important to understand the mechanisms underlaying trained immunity if there is to be a potential for the future development of novel therapeutic strategies to prevent and treat allergic diseases (48, 112). Trained immunity is seen as a possible mechanism to explain the non-specific effects associated to some vaccines based on attenuated and/or inactivated pathogens that trigger pattern recognition receptors (PPR) (113). Trained immunity-based vaccines confer a broad protection by impacting innate immune cells and promoting non-specific response, while modulating specific adaptative T cell responses (30, 114). For example, early life vaccination with BCG has been linked with an enhanced Th1 immune response (115) and downmodulation of the Th2 allergic phenotype (116). However, other authors could not associate BCG vaccination with protection from allergic airway disease (117, 118) or suspected sensitization/food allergy (119). Another study found that whole cell killed pertussis vaccine was associated with protection against allergic disorders, including food allergy (120). Thus, a pioneering use of trained immunity could be related to its applicability in allergic prevention and treatment, acting as an immune system regulator. Recent investigations suggest that generation of novel vaccines with allergens for allergen specific immunotherapy (AIT), could imprint a tolerogenic phenotype by promoting metabolic and epigenetic reprogramming; and restore levels of ILCs, monocytes, dendritic cells and macrophages contributing to the establishment of a healthy immune responses to allergens (31, 121, 122). As well, new findings related to sublingual and subcutaneous AIT showed that the prolonged exposition to allergen induces the differentiation of naïve ILC2s into IL-10^+^ILC2 cells, which contribute to immune tolerance by restoring epithelial cell barrier integrity and repressing allergic Th2 responses (123, 124). Traditionally the effectiveness of AIT to treat IgE-mediated allergies was associated with increments in the production of allergen-specific IgG antibodies that shifts the recognition of allergens away from IgE, reducing the symptoms of allergic inflammation with long-lasting effects (112). For example, in a peanut-sensitized mice model, immunotherapy with virus-like particle (a molecular form of AIT) coupled to a single peanut allergen induced specific IgG antibodies, that conferred protection against anaphylaxis after systemic challenge with the whole peanut extract and diminished local reactions after skin prick tests (125). AIT also induce regulatory T and B cells, suppress Th2 and skew towards Th1 cells differentiation (123). However, the capacity of next generation of AIT allergen vaccines to restore/modulate innate immune responses in addition to regulate adaptative immune responses needs to be definitively demonstrated. The use of AIT as a preventive and/or therapeutic approach to reduce allergen-induced symptoms during allergic inflammatory diseases have been review elsewhere (112). Overall, understanding how AIT-induced trained immunity to reestablish innate immune homeostasis may lead to the use of AIT as a preventive strategy in allergic diseases.

## 5. Final thoughts

**Figure 2:**
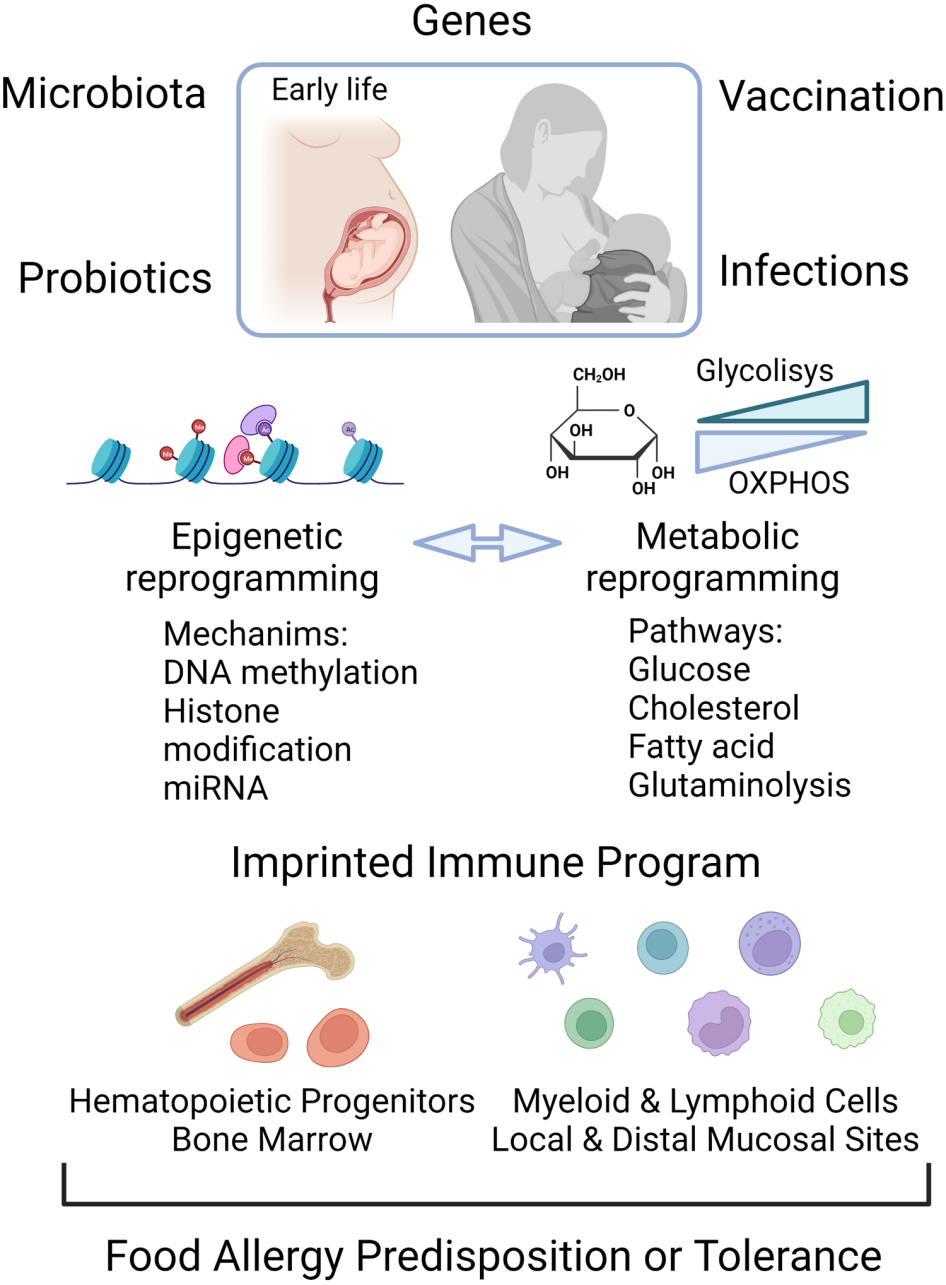
Epigenome and metabolic modification early in life are determining factors for the establishment of food allergy or for a tolerant trained immune phenotype. Food allergy can be triggered by the interaction of genes with the environment. Early life exposures to microbial infection, vaccines, probiotics and altered gut microbiota can shape the epigenetic and metabolic program, the components of which can be reciprocally regulated. The control of the transcriptional landscape by DNA methylation, histone modifications and non-coding RNA molecules, as well as changes in the energetic metabolic, leads to activation of local immune cells and hematopoietic progenitors in bone marrow as functional trained cells. Created with Biorender.

Initial studies have implicated trained immune responses in the establishment, development and persistency of food allergy. Growing evidence indicates that early life epigenetic and metabolic reprograming underlies the increased responsiveness to food allergens. The changes in cellular metabolism and the epigenetic program contribute to the regulation of the immune function and correlate with clinical outcomes (Figure 2). Longitudinal studies are needed to characterize the temporal relationship of the epigenetic and metabolic status of diverse immune cells population in this allergic disease. This approach will help to validate the potential efficacy and optimal use of innate cell therapy for regulating the frequency and/or activation status as an accurate preventive strategy in food allergy. In addition, the use of probiotics to selectively modulate intestinal microbiota to produce dynamic changes in the epigenetic landscape and metabolic pathways that regulates the immune response may be useful for altering food allergy development and severity. More studies are needed to elucidate if the protective and immunomodulatory effects in different mucosal sites reported for probiotic supplementation are related to an effective trained immune response, and how understanding of this response may be used in the prevention and treatment of anaphylactic food allergy.
